# Administration of *Ginkgo biloba* Extract (EGb761) Alone and in Combination with FK506 Promotes Liver Regeneration in a Rat Model of Partial Hepatectomy

**DOI:** 10.4274/balkanmedj.2016.1830

**Published:** 2018-03-15

**Authors:** Nahide Ekici Günay, Sabahattin Muhtaroğlu, Abdulkadir Bedirli

**Affiliations:** 1 Department of Clinical Biochemistry, University of Health Sciences, Training and Research Hospital, Kayseri, Turkey; 2 Department of Clinical Biochemistry, Erciyes University School of Medicine, Kayseri, Turkey; 3 Department of General Surgery, Gazi University School of Medicine, Ankara, Turkey

**Keywords:** EGb761, FK506, hepatectomy, liver, oxidative stress, regeneration, thymidine kinase, mitosis

## Abstract

**Background:**

Free radical damage is known to occur during liver regeneration. The *Ginkgo biloba* extract EGb761 has antioxidant properties due to its ability to scavenge free radicals. FK506 has been widely used as an immunosuppressant that stimulates hepatocyte proliferation following partial hepatectomy.

**Aims:**

To explore whether EGb761 enhances liver regeneration after hepatectomy in rats, we investigated the effects of EGb761 alone and in combination with FK506 on the liver regenerative process.

**Study Design:**

Animal experimentation.

**Methods:**

A total of 75 Wistar albino rats weighing 340.08±11.66 g were randomly divided into five experimental groups: sham, control, FK506, EGb761, and FK506 + EGb761. According to the study groups, rats were administered FK506 at a dose of 0.1 mg/kg/day and EGb761 at 25 mg/kg/day three times via the intraperitoneal route. Then, two-thirds hepatectomy was performed according to the Higgins and Anderson technique in all the rats. At postoperative 48 h, 53 surviving rats were sacrificed. Serum and plasma samples were collected for analyzing thymidine kinase and oxidative stress marker levels. The regenerated liver was entirely resected, weighed, and sectioned. The mitotic index was assessed using hematoxylin-eosin staining. The extent of liver regeneration was calculated using the Child’s formula. The data were statistically analyzed using ANOVA, with a significance level of 5% (p<0.05).

**Results:**

Rats who received EGb761 showed significantly higher levels of liver regeneration than those who received FK506 or FK506 + EGb761 (p<0.01). Thymidine kinase level and mitotic index were significantly higher in the EGb761 (p<0.005) and FK506 (p<0.05) groups than in the control and sham groups. In addition, the liver regeneration percentage was significantly higher in the EGb761 group than in the FK506 group (p<0.01). Myeloperoxidase and malondialdehyde levels were significantly correlated between the EGb761 and FK506 groups, even at lower levels in the EGb761 group (p<0.001).

**Conclusion:**

EGb761, which is an antioxidant, reduces liver damage and stimulates liver regeneration following partial hepatectomy in rats through its anti-inflammatory and antioxidative effects.

Liver regeneration following partial hepatectomy is regulated by the immune system (1). In the early phase of liver regeneration, free radical damage is known to occur; lipid peroxidation has been demonstrated in liver homogenates during the early phase of liver regeneration (2). Extracts of *Ginkgo biloba* leaves contain approximately 300 chemicals. While the precise role of each component remains unclear, studies have shown that flavonoids and terpenoids are the most effective components in the EGb761 extract. The standardized formulation of *G. biloba* extract comprises approximately 24% of flavonoid glycosides (primarily quercetin, kaempferol, and isorhamnetin) and 6% of terpene lactones (2.8%-3.4% of ginkgolides A, B, and C and 2.6%-3.2% of  bilobalide) (3). EGb761 is known for its antioxidant properties due to its ability to scavenge free radicals and neutralize ferryl ion-induced peroxidation (4). It can antagonize platelet-activating factor in rats (5). The administration of EGb761 increases arterial and capillary blood flow in ischemic vascular disease. Particularly, terpenoids and flavonoids from EGb761 possess vasorelaxant properties. Furthermore, EGb761 enhances nerve regeneration in the central and peripheral nerve systems (6). On the other hand, FK506 (tacrolimus), which has been widely used as an immunosuppressant, is a potent calcineurin inhibitor that weakens the effects of cytokines, thereby stimulating hepatocyte proliferation following partial hepatectomy (7). Recently, herbal medications have been increasingly considered as an effective and safe reinforcement to synthetic drugs. However, there is a lack of approval for these herbal medications due to inadequate research conducted using experimental liver regeneration models. Therefore, in this study, we investigated whether EGb761, with and without FK506, promotes liver regeneration in a rat model for partial hepatectomy.

## MATERIALS AND METHODS

The experimental protocol was approved by the animal research ethics committee of Erciyes University, Kayseri, Turkey (Approval no: 2004-a18).

### Chemicals

Reduced glutathione, NADH disodium salt, nitrobluetetrazolium, phenazine methosulphate, thiobarbituric acid, p-nitrophenyl phosphate, trichloroacetic acid, chloroform, sodium pyrophosphate, Triton X-100, hydrogen peroxide, and methanol were procured from Merck Specialties and Sigma-Aldrich Chemicals (St. Louis, MO). Solvents used for thin layer chromatography, extraction, and estimation of oxidative stress were of analytical grade. FK506 monohydrate was purchased form Astellas Pharma US Inc. (Tokyo, Japan) and EGb761 from Sigma-Aldrich (Catalog number NIST SRM 3246). Thymidine kinase activity was radioimmunologically measured using the Prolifigen^®^ thymidine kinase (TK)-radioenzymatic assay (REA) commercial kit (Sangtec Medical, Sweden).

### Rats and treatments

Male Wistar albino rats aged 12 weeks and weighing 300-400 g were supplied by the Animal Experimental Center of Erciyes University (Turkey). The rats were kept under controlled conditions of temperature (22-24 ºC) and humidity (40%±10%) with a 12 h light/dark cycle. The rats had access to commercial rodent food and water *ad libitum*. All procedures involving animals were conducted in strict accordance with the Turkish legislation on the use and care of laboratory animals and guidelines established by the Institute for Experimental Animals of Erciyes University (Turkey). A total of 75 rats were divided into five groups: the sham group underwent excision of only the hepatic suspensory ligament, whereas the control (5 mL/kg of 0.9% NaCl), EGb761, FK506, and EGb761 + FK506 groups underwent hepatectomy. EGb761 (25 mg/kg, i.p.), FK506 monohydrate (1 mg/kg, i.p.), and combination of these drugs were administered once daily for three consecutive days to the rat medicament groups. After 3 days, the rats underwent 70% hepatectomy according to the Anderson and Higgins technique based on the calculation of liver regeneration volume using the Child’s formula. Partial hepatectomy was performed under anesthesia (10/90 mg/kg xylazine/ketamine i.p.) as previously reported (8). A resection of 70% was selected because it is the critical limit for liver failure (9).

### Biochemical measurements

The rats were killed 48 h after hepatectomy, and blood samples were collected from the hepatic veins. The protein content in the liver homogenate was analyzed according to the method of Lowry et al. (10). Bovine serum albumin (BSA) (1 mg/mL) was used as a standard. The protein content in the samples was estimated from a standard curve obtained using different BSA concentrations. The activities of aspartate aminotransferase (AST), alanine aminotransferase (ALT), and gamma glutamyl transferase (GGT), alkaline phosphatase (ALP) and lipid profiles were assayed using a Konelab 60i automatic analyzer (Thermo Fisher Scientific, Finland).

### Calculation of the liver regeneration index

The excised wet liver weight was recorded at the time of hepatectomy. The liver weight as a percentage of the body weight was expressed as a relative liver weight (RLW) to compare the rates of liver regeneration between the groups. To calculate RLW, the residual liver weight after partial hepatectomy was subtracted from the liver weight measured at the time of sacrifice. Then, a ratio of this value to the total liver weight [accepted as 3.5% of the rat body weight (11)] was calculated and multiplied by 100 to yield the liver regeneration rate. The liver regeneration rate was calculated according to the Child’s formula (Equation 1) (12). 

The results are expressed as percentages. The remaining liver which included regenerated tissue and blood specimens collected from surviving and sacrificed rats (n=51) after 18 h were placed in tubes and stored at -80 °C until further use.

### Oxidative stress estimation

Antioxidant activity was evaluated in the liver tissue and serum. To calculate antioxidant activity in liver samples, tissues were collected in screw-capped polypropylene vials and homogenized in ice-cold 0.1 M Pharmaceutical Benefits Scheme (PBS) (pH 7.4) at a ratio of 10:1 (100 mg of tissue in 1 mL of PBS). After centrifugation at 10.000 rpm for 30 min at 4 ºC, the supernatant was collected. To assess oxidative stress, activities of thiobarbituric acid reactive substances were measured using the method described by Okhawa et al. (13). The results are expressed as nmol/mL of malondialdehyde (MDA) production per gram of the wet tissue. Myeloperoxidase enzyme activity was estimated using the method described by Bradley et al. (14), and the increase in absorbance at 460 nm was measured using spectrophotometry. The superoxide dismutase (SOD) (15), glutathione peroxidase (16), and glutathione reductase assays were performed using the methods previously described (17).

### Histopathological examination

Regenerated liver tissue specimens were fixed in formaldehyde and stained with hematoxylin-eosin. The ratio of the number of hepatocytes undergoing mitosis to the total number of hepatocytes was reported as the mitotic index (MI).

### Statistical analysis

All numerical data are reported as the mean ± standard deviation. Statistical analysis was performed using the SPSS 22.0 software for Windows. The assumption of normality was checked using the Kolmogoro-Smirnov test. Group comparisons were analyzed using ANOVA, and pair-wise comparisons were performed using the Scheffe post-hoc test. Statistical significance was defined as p<0.05.

## RESULTS

**Figure 1:**
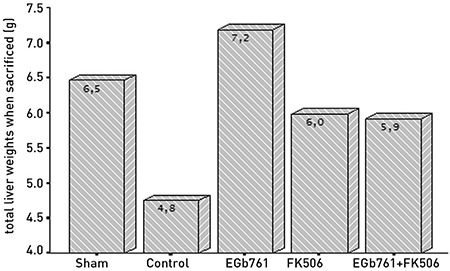
Total liver weights of sacrificed rats according to study groups.

**Figure 2:**
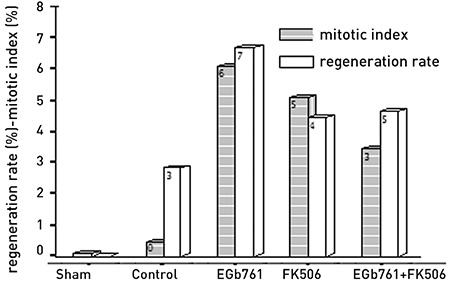
Graph of regeneration rate and mitotic index according to study groups.

**Figure 3:**
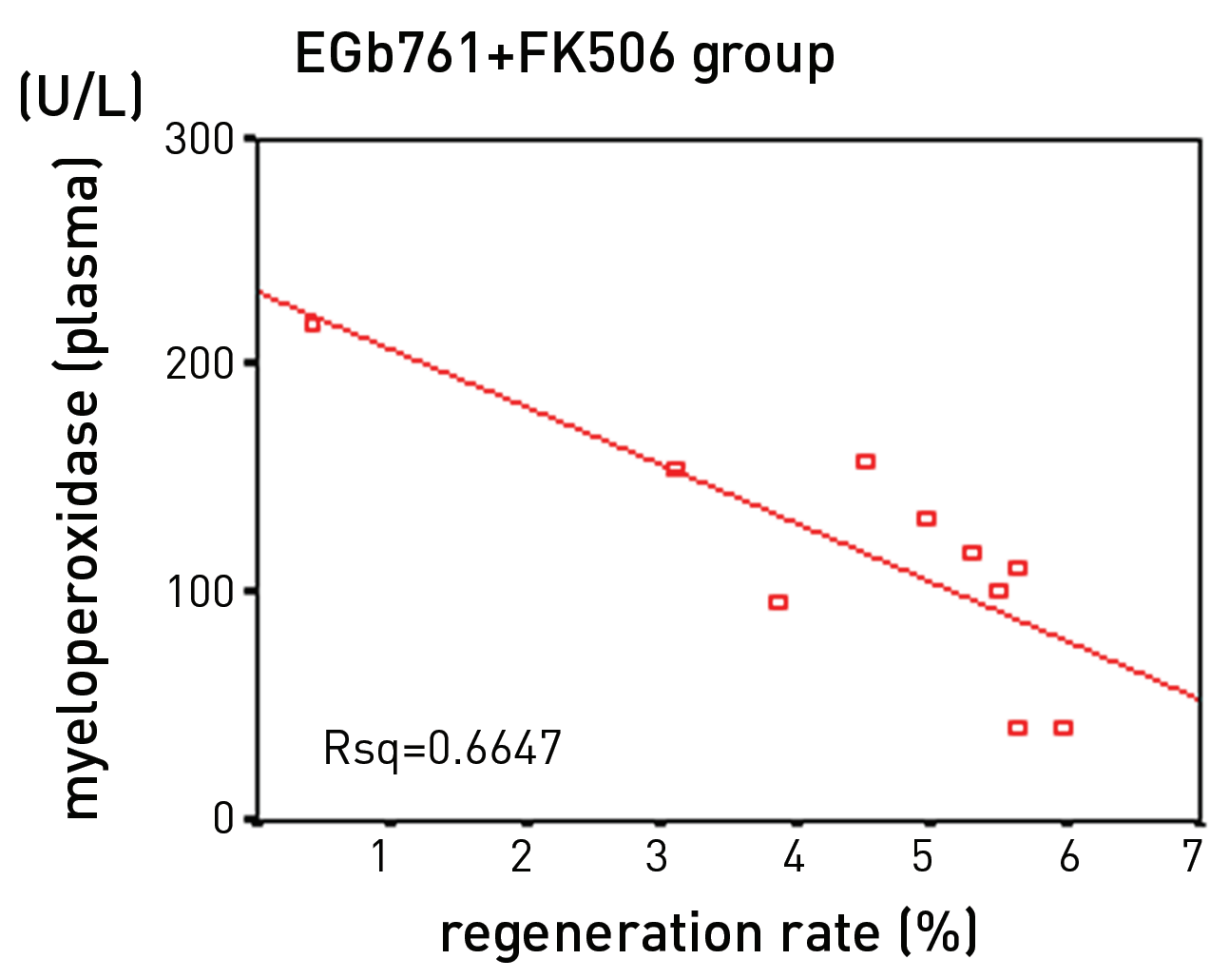
Myeloperoxidase and regeneration rate correlation curve.

In the present study, the liver regeneration ratio, MI, extent of liver damage, and oxidative stress marker levels were investigated after the administration of EGb761 in a rat hepatectomy model. For this purpose, the effect of EGb761 was compared with that of the immunosuppressant FK506, which is widely used and has a well-known regenerative effect on the liver. None of the rats died during the course of drug administration prior to hepatectomy. In the period between hepatectomy and sacrifice, 22 rats died (three in the sham, five in the control, four in the EGb761, five in the FK506, and five in the EGb761 + FK506 groups) and were excluded from the study. The liver regeneration parameters (thymidine kinase, regeneration rate, mitotic index) and antioxidant enzyme levels (glutathione reductase, myeloperoxidase, MDA levels) were showed to be significant differences among the sham, control, FK506 and EGb761 + FK506 groups (p<0.05). Moreover, the EGb761 group had higher liver regeneration rate and MI than those in the FK506 and EGb761 + FK506 groups. The liver weights, regeneration rates, and mitotic indices of the groups are shown in Table 1 and Figures 1,2,3,4a,4b,4c,4d,4e, respectively.  The EGb761 group showed higher levels of ALT and AST than the sham group (p<0.05). However, there were no significant differences in these levels among the four groups that underwent hepatectomy. The EGb761, FK506, and EGb761 + FK506 groups had significantly higher serum ALP levels than those in the control and sham groups (p<0.05), although the increase in the EGb761 group was not as high as in the other drug treatment groups. There was no significant difference among the groups in terms of GGT levels. The serum parameters are summarized in Table 2.

There were significant differences between the groups in terms of thymidine kinase levels, with highest levels in the EGb761 and FK506 groups, followed by the EGb761 + FK506 group (p<0.05) (Table 3). The mitotic indices of the EGb761 and FK506 groups were significantly higher than those of the control and sham groups (p<0.001). Liver histology slides are shown in Figures 4a,4b,4c,4d,4e. The antioxidant enzyme levels are summarized in Table 4. The SOD levels did not differ between the groups, whereas MDA levels in tissue samples were significantly lower in the EGb761 + FK506 group than in the FK506 group. Additionally, MDA levels were significantly lower in the EGb761 group than in the FK506 group. The correlations were studied in each rat group. There was a positive correlation between thymidine kinase with triglyceride (r=0.78, p<0.05) and liver regeneration rate (r=0.73, p<0.05). In the EGb761 + FK506 group, there was a negative correlation between the plasma MPO level and liver regeneration rate (r=-0.81, p<0.05) (Figure 3).

## DISCUSSION

Regeneration is a vital process, which is expected to occur after partial hepatectomy and liver transplantation. The present study revealed that the administration of 25 mg/kg of EGb761 alone or in combination with FK506 increased the liver regeneration rate, MI, and regenerated liver mass in a rat partial hepatectomy model.

The hepatic regenerative process in rats begins with an increase in the levels of various signaling molecules from the third hour after hepatectomy, peaks within 2-3 days, and ends at 5-7 days when the remnant lobe enlarges to the size of the original liver (18). In a similar study of rat liver regeneration after partial hepatectomy, it was shown that maximum liver regeneration at 36 and 48 h was significantly higher, even when only one drug dose (ICI 182,780) was administered 24 h before hepatectomy (19). In another rat study, orthotropic liver transplantation was performed after partial hepatectomy, with EGb761 intravenously administered 1 h prior to surgery, resulting in alleviation of hepatic inflammation/necrosis between 2 and 24 h postoperatively (20). In our study, the rats were sacrificed at the 48th hours when the liver regeneration rate was expected to be the maximum. The actual and expected regeneration rates in our study were consistent for all three drug doses. Flavonoids are extensively metabolized following oral administration and undergo metabolic transformation, such as methylation, sulfation, and glucuronidation, thereby changing their structures and biological activities. Oral administration of EGb761 may have a prolonged effect (up to 8 weeks), and doses up to 100 mg/kg have been used for various chronic and degenerative diseases, such as cancers and cardiovascular diseases, in both* in vitro* and *in vivo *study models (21). EGb761 has a broad range of therapeutic effects, and low doses have also yielded beneficial effects in some experimental models. In animal trials, the pharmacological activity of EGb761 was found to be significantly better with intraperitoneal administration than with oral administration, even at low (10 mg/g) doses for neurological recovery (22). Therefore, we used the intraperitoneal administration route in our study due to its effective pharmacological effect. Severe liver damage can occur in many cases, such as ischemia, followed by reperfusion, resection, and transplantation. The methods of reducing the damage caused by ischemia-reperfusion in the liver have been studied. FK506 is known to be an immunosuppressant that ameliorates hepatic damage due to ischemia and reperfusion in rats (23). The inactivation of free radicals, which are mainly responsible for tissue damage, reduces inflammation and accelerates the regenerative process (24). In this context, we used EGb761 along with the free radical scavenger FK506. We aimed to investigate the use of an antioxidant along with an immunosuppressant, such as FK506. One of the main results of our study was that EGb761, by reducing tissue oxidative stress, positively contributed to the regeneration process. We could not find studies on the effectivity of EGb761 alone or in combination with FK506 on liver injury in a rat hepatectomy model. The combination of EGb761 and FK506 had a considerable effect on the liver regeneration rate. Furthermore, EGb761, when used alone, showed a regenerative effect, which was similar to that of FK506. This indicated that the effect of EGb761 and FK506 in stimulating liver regeneration was synergistic. Drugs with different mechanisms may be more effective for this purpose when used together. These results are consistent with those of a previous report which stated that the administration of EGb761 promoted peripheral nerve regeneration and neovascularization in rats (25). Although all the groups showed higher results than those of the sham group, EGb761 and FK506 administration did not affect AST and ALT levels in the hepatectomy model. The lack of difference between the groups in terms of GGT levels can be explained by the fact that GGT, which can be induced by some drugs, is unaffected by EGb761. FK506 has been reported to increase ALP levels in osteoblasts with proliferative effects (26), but we could not find a similar study on EGb761. It is known that ALP levels are elevated during liver regeneration (27). Because the control group also underwent hepatectomy, we could not associate elevated ALP levels (an average increase of 216% in all the groups) with hepatectomy. Therefore, elevated ALP levels were attributed to FK506 and EGb761 administration. The evaluation of ALP levels after FK506 or EGb761 administration, either alone or in combination, can be used for reflecting the positive proliferative effect of these drugs on the liver cells in the regeneration process. Some studies have indicated that the levels of SOD, an antioxidant enzyme, are low after partial hepatectomy, although others have not found such an effect. When EGb761 was used in rats with liver fibrosis induced by intraperitoneal injection of carbon tetrachloride, there was an increase in the SOD levels (28); however, we did not observe a significant increase in the SOD levels in our model. Furthermore, we did not find any other study in which the association between EGb761 and SOD levels was investigated using the same model. After hepatectomy, free radicals generated as a result of lipid peroxidation on the hepatic cell membrane cause tissue damage. The best way to assess lipid peroxidation is to measure MDA levels (2). According to our findings, combined administration of FK506 and EGb761 had a synergistic effect, resulting in significantly lower MDA levels (p<0.001). Thymidine kinase can be measured in the blood of hepatectomized rats, providing a noninvasive assessment of liver regeneration (29). We found that thymidine kinase levels in the EGb761 and FK506 groups were significantly higher than in the control and sham groups (p<0.001), with similar results for the regeneration rate (%) and MI (p<0.001). However, we did not find any study on the association between thymidine kinase levels, regeneration rate, and MI as measures of liver regeneration after EGb761 and FK506 administration, either alone or in combination with each other, in a rat partial hepatectomy model. Therefore, we could not perform a comprehensive comparison between the results obtained in our study and those in other studies in terms of the effect of EGb761 in a rat hepatectomy model.

We showed that EGb761 administration before hepatectomy in rats reduces the extent of tissue damage caused by free radicals and inflammation. EGb761 administration also induces liver regeneration by reducing the extent of liver damage through its antioxidative and anti-inflammatory effects in a rat hepatectomy model. Further clinical examinations are necessary, and detailed experiments should be performed for the use of EGb761 in the treatment of patients after hepatectomy and liver transplantation.

## Figures and Tables

**Table 1:**
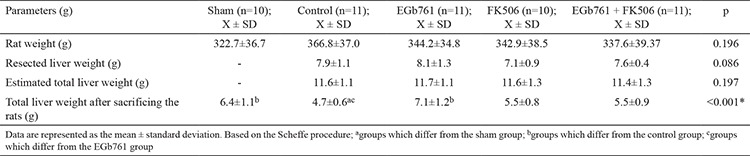
Liver weights of rats

**Table 2:**
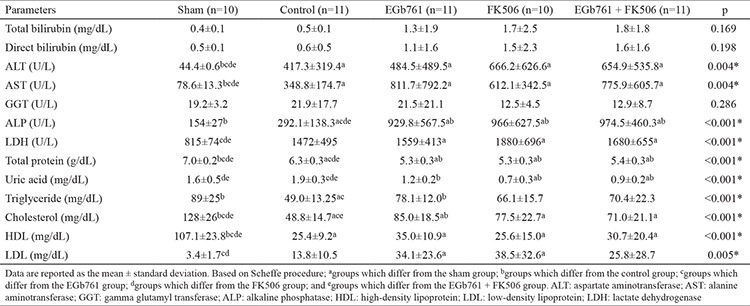
Biochemical parameters of rats

**Table 3:**
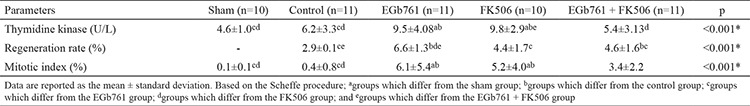
Liver regeneration parameters

**Table 4:**
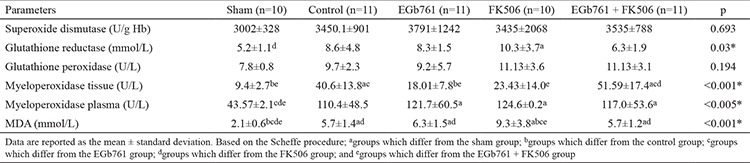
Antioxidant enzyme levels in rats

**Equation 1:**
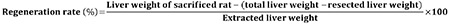
Liver regeneration rate’s (equation 1) expression by words math application;

**Figure 4a:**
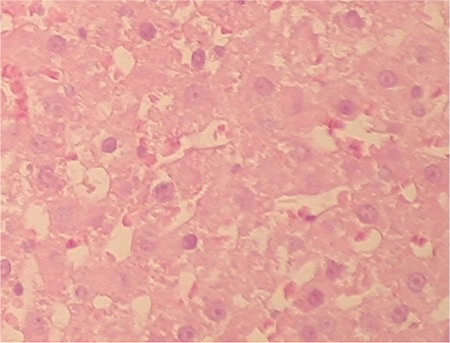
Photomicrograph of liver sections of sham group rat 48 h after a 70% partial hepatectomy. Note the absance of mitotic figures in the hepatocytes (H&E x20).

**Figure 4b:**
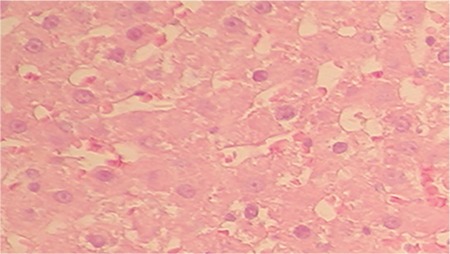
Photomicrograph of liver sections of control hepatectomy group rat 48 h after a 70% partial hepatectomy. Note the absance of mitotic figures in the hepatocytes (H&E x20).

**Figure 4c:**
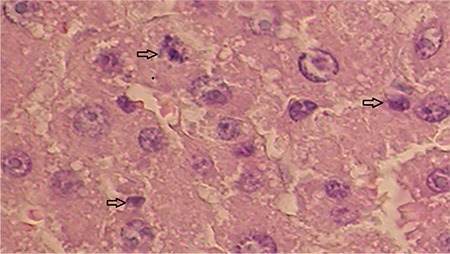
Photomicrograph of liver sections of FK506 group rat after 48 h a 70% partial hepatectomy. The arrows denote multiple mitotic figures in the hepatocytes (H&E x20).

**Figure 4d:**
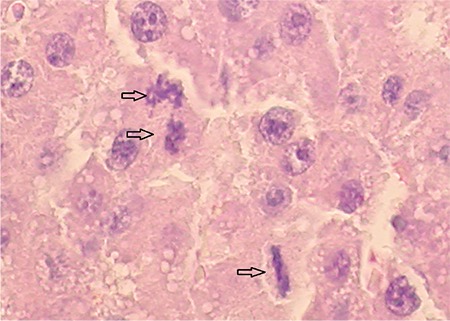
Photomicrograph of EGb761 group rat's liver sections after 48 h a 70% partial hepatectomy. The arrows denote multiple mitotic figures. Note intensive nuclear enlargement in the hepatocytes (H&E x20).

**Figure 4e:**
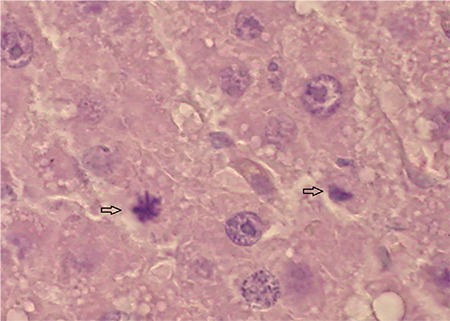
Photomicrograph of liver sections of drug combination group (EGb761 + FK506) rat after 48 h a 70% partial hepatectomy. The arrows denote mitotic figures in hepatocytes (H&E x20) group.
